# Possible Association of Papillophlebitis with Guillain-Barré Syndrome: Case Report

**DOI:** 10.4274/tjo.98522

**Published:** 2018-09-04

**Authors:** Müge Çoban Karataş, Merih Soylu

**Affiliations:** 1 Başkent University Faculty of Medicine, Department of Ophthalmology, Ankara, Turkey; 2 Private Practice, Adana, Turkey

**Keywords:** Papillophlebitis, Guillain-Barré syndrome, visual deterioration

## Abstract

In this case report, we presented a patient with visual deterioration as a result of papillophlebitis in the right eye who was later diagnosed with Guillain-Barré syndrome (GBS). Upon systemic and laboratory work-up to determine the etiology of papillophlebitis, the diagnosis of GBS was made and treatment was initiated immediately. The ocular and systemic symptoms resolved quickly after starting intravenous immunoglobulin therapy. We present this case to emphasize the importance of etiological diagnosis in papillophlebitis and the unusual presentation of GBS.

## Introduction

Papillophlebitis is an uncommon ocular condition of undetermined etiology. Unlike classic central retinal vein occlusion, patients suffering from this disease are usually healthy and younger than 50 years of age.^1,2^ Most patients complain of blurred vision and photopsia. Typical findings include dilatation and tortuosity of the major retinal veins with retinal hemorrhage and optic disc edema.^2^ Traditional treatment for papillophlebitis includes systemic and periocular steroid therapy, intravitreal triamcinolone, intravitreal anti-VEGF inhibitors, platelet inhibitors, and anticoagulation.^3,4,5^ Guillain-Barré syndrome (GBS) is an immune-mediated acute polyneuropathy principally affecting motor nerves and causing paralysis.^6^ It is the most common cause of acute muscle weakness associated with peripheral neuropathy in adults and can be lethal if not treated early.^7^ GBS is reported to be associated with Zika virus infection.^8^ There are a few case presentations in the literature reporting total ophthalmoplegia, optic nerve involvement, ptosis, Vogt-Koyanagi-Harada, and uveitis as ocular findings of GBS.^9,10,11,12^

Here we report a case with visual deterioration in the right eye with numbness, pain, and tingling sensation in both lower legs.

## Case Report

**Figure 1:**
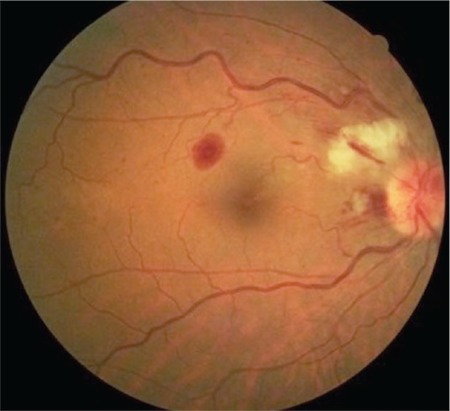
Fundoscopic examination revealed splinter hemorrhages, optic nerve head hemorrhage, and cotton wool spots due to ischemia in the superior arcuate region and a diagnosis of papillophlebitis was made

**Figure 2:**
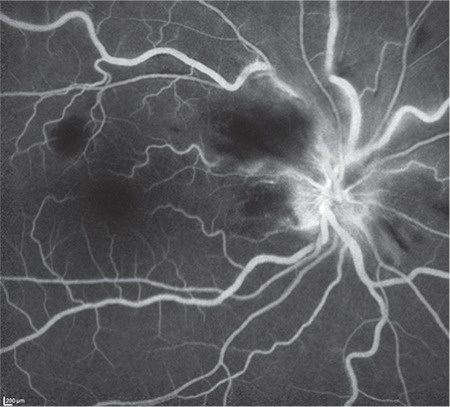
Fundus fluorescein angiography revealed no ischemic areas; however, there was hypofluorescence in the areas corresponding to hemorrhages and hyperfluorescence in the optic nerve head

**Figure 3:**
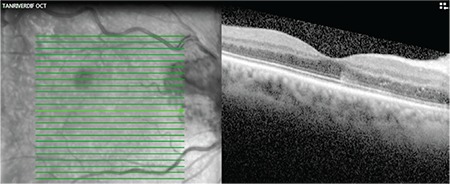
Optical coherence tomography revealed macular edema and intraretinal edema and hyperreflective spots in the nasal fovea corresponding to the areas affected by the occlusion

**Figure 4:**
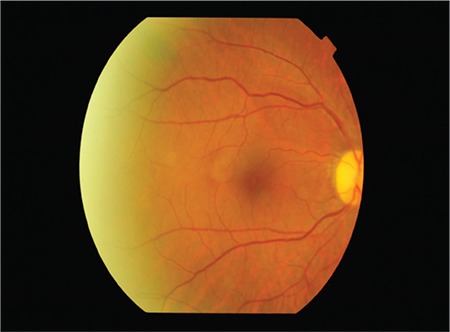
During follow-up, the patient’s best corrected visual acuity in the right eye returned to 1.0 without any treatment for ocular findings. Hemorrhages and cotton wool spots improved without any specific ocular treatment

A 53-year-old woman presented with complaints of visual deterioration in the right eye. Her anamnesis revealed no ocular or systemic diseases except a mild influenza-like illness a week earlier. Her best corrected visual acuity (BCVA) was 0.5 in the right and 1.0 in the left eye. Anterior segment examination and intraocular pressure was within normal range in both eyes. Fundoscopic examination of the right eye revealed splinter hemorrhages, optic nerve head hemorrhage, and cotton wool spots in the superior arcuate region, and the patient was diagnosed with papillophlebitis (Figure 1). Fundus fluorescein angiography revealed no ischemic areas; however, there was hypofluorescence in the areas corresponding to hemorrhages, and hyperfluorescence in the optic nerve head (Figure 2). Optical coherence tomography revealed macular edema and intraretinal edema and hyperreflective spots in the nasal fovea corresponding to the areas affected by the occlusion (Figure 3). Laboratory and radiological tests were requested to determine the etiology of the papillophlebitis. One week after onset of these complaints, the patient began to experience numbness, pain, and tingling sensation in both lower legs. Motor weakness became progressively severe in both extremities and she was admitted to the neurology clinic for advanced examination and treatment. No abnormalities were detected in magnetic resonance imaging of the brain and spinal cord. Complete blood count, electrolytes and blood chemistry and urinalysis were normal. Coagulation tests, including serum levels of homocysteine, protein C and S, partial thromboplastin time, and prothrombin time were normal. Erythrocyte sedimentation rate and anticardiolipin G and M were within normal range. Lumbar puncture revealed no pathology. She was diagnosed with GBS and treated with intravenous immunoglobulin (IVIg) therapy. Her symptoms improved in the following 3 months. During follow-up, her BCVA in the right eye returned to 1.0 without any treatment for ocular findings (Figure 4).

## Discussion

GBS is usually preceded by infection or other immune stimulation that induces an aberrant autoimmune response targeting the peripheral nerves and their spinal roots.^13,14^ Two-thirds of adult patients report preceding symptoms of a respiratory or gastrointestinal tract infection within 4 weeks of onset.^15^ Underlying systemic diseases such as systemic lupus erythematosus (SLE), sarcoidosis, Hodgkin disease, and other neoplasms have been known to cause a small number of GBS cases.^16^

The pathogenesis of GBS as a manifestation of active SLE is not clear, but both cell-mediated and humoral processes may play a significant role.^17^ Ocular findings in SLE include hemorrhage, retinal cotton wool spots, microangiopathy, and vaso-occlusion as a result of immune complex deposition. The role of immune complex deposition is highlighted in vascular pathogenesis in the eye.^15^

Our patient was relatively young and did not suffer from any systemic diseases. Her anamnesis revealed only an influenza-like illness with mild symptoms the week before. To the best of our knowledge, there are no previous reports in the literature of papillophlebitis as the initial presentation of GBS. Although the two clinical presentations may be coincidental, it may be postulated that papillophlebitis in our patient was related to the immune-mediated etiology of GBS, as is seen in SLE.

In conclusion, we present this case in order to emphasize the importance of etiologic diagnosis in papillophlebitis. Papillophlebitis may be an initial finding of GBS, which may lead to serious neurologic complications if not treated early. Treatment modalities may differ with the etiologic diagnosis. Early initiation of IVIg or plasma exchange is of proven benefit and crucial in GBS.^19^ In our patient, no specific treatment for ocular findings was applied, as the papillophlebitis resolved with systemic treatment of GBS without leaving any sequelae. This may also be the result of the benign course of the papillophlebitis and the reversal of the findings of papillophlebitis may be the natural outcome.
